# Functional Evaluation of 3D Liver Models Labeled with Polysaccharide Functionalized Magnetic Nanoparticles

**DOI:** 10.3390/ma15217823

**Published:** 2022-11-05

**Authors:** Yoshitaka Miyamoto, Yumie Koshidaka, Katsutoshi Murase, Shoichiro Kanno, Hirofumi Noguchi, Kenji Miyado, Takeshi Ikeya, Satoshi Suzuki, Tohru Yagi, Naozumi Teramoto, Shuji Hayashi

**Affiliations:** 1 Department of Reproductive Biology, National Research Institute for Child Health and Development, 2-10-1 Okura, Setagaya-ku, Tokyo 157-8535, Japan; 2 Department of Advanced Medicine in Biotechnology and Robotics, Nagoya University Graduate School of Medicine, 65 Tsurumai-cho, Showa-ku, Nagoya 466-8550, Japan; 3 Department of Mechanical Engineering, Tokyo Institute of Technology, 12-2-1 Ookayama, Meguro-ku, Tokyo 152-8552, Japan; 4 Department of Applied Chemistry, Faculty of Engineering, Chiba Institute of Technology, 2-17-1 Tsudanuma, Narashino, Chiba 275-0016, Japan; 5 Life Science Research Center, University of Toyama, 2630 Sugitani, Toyama 930-0194, Japan; 6 Nagoya Research Laboratory, Meito Sangyo Co., Ltd., 25-5 Kaechi, Nishibiwajima, Kiyosu, Aichi 452-0067, Japan; 7 Department of Regenerative Medicine, Graduate School of Medicine, University of the Ryukyus, Nishihara-cho, Okinawa 903-0215, Japan; 8 Photosensitive Materials Research Center, Toyo Gosei Co., Ltd., 4-2-1 Wakahagi, Inzai-shi, Chiba 270-1609, Japan; 9 Research Laboratories, HAB Research Organization, Ichikawa General Hospital, 5-11-13 Sugano, Ichikawa, Chiba 272-8513, Japan

**Keywords:** contrast agents, magnetic iron oxide nanoparticles, cell labeling, 3D culture, primary hepatocytes, endothelial cell, spheroid, liver function, functional evaluation

## Abstract

Establishing a rapid *in vitro* evaluation system for drug screening is essential for the development of new drugs. To reproduce tissues/organs with functions closer to living organisms, *in vitro* three-dimensional (3D) culture evaluation using microfabrication technology has been reported in recent years. Culture on patterned substrates with controlled hydrophilic and hydrophobic regions (Cell-able^TM^) can create 3D liver models (miniature livers) with liver-specific Disse luminal structures and functions. MRI contrast agents are widely used as safe and minimally invasive diagnostic methods. We focused on anionic polysaccharide magnetic iron oxide nanoparticles (Resovist^®^) and synthesized the four types of nanoparticle derivatives with different properties. Cationic nanoparticles (TMADM) can be used to label target cells in a short time and have been successfully visualized *in vivo*. In this study, we examined the morphology of various nanoparticles. The morphology of various nanoparticles showed relatively smooth-edged spherical shapes. As 3D liver models, we prepared primary hepatocyte–endothelial cell heterospheroids. The toxicity, CYP3A, and albumin secretory capacity were evaluated in the heterospheroids labeled with various nanoparticles. As the culture period progressed, the heterospheroids labeled with anionic and cationic nanoparticles showed lower liver function than non-labeled heterospheroids. In the future, there is a need to improve the method of creation of artificial 3D liver or to design a low-invasive MRI contrast agent to label the artificial 3D liver.

## 1. Introduction

Establishing a rapid *in vitro* evaluation system for drug screening is essential for the development of new drugs [1,2,3]. The liver is an essential organ *in vivo*, and many attempts have been made to artificially mimic the liver *in vitro* [4,5,6,7,8]. These cell culture techniques are used in many fields including drug discovery, medicine, and cosmetics. For example, two-dimensional (2D) culture via a substrate or extracellular matrix (ECM) is simple and effective for studying cell interactions with drugs [9,10] and is used for screening in drug development. However, they exhibit behavior that differs from that of cell proliferation and morphology. To reproduce tissues and organs with functions that are similar to those of living organisms, *in vitro* evaluation using three-dimensional (3D) culture has been conducted in recent years [4,5,6,7,8,11,12]. Specifically, it has been reported that 3D culture is superior to 2D culture in terms of tissue/organ structure, cell differentiation ability, proliferation, drug toxicity, and metabolism, with results closer to those obtained *in vivo* [5,13,14,15].

It is necessary to determine the tissue/organ using 3D culture and select cells and culture techniques that are appropriate to the conditions. There are a variety of 3D culture techniques, including spheroid and organoid cultures [4,5,6,7,8,11,12,13,14,16,17,18,19], agitated culture in a bioreactor [20], hydrogel culture (ECM and synthetic polymers) [21], and cell sheet culture [22,23,24]. Whitesides et al. found that hepatocyte morphology and function could be controlled using microfabrication techniques [25,26]. By creating patterned substrates with controlled hydrophilic and hydrophobic regions by microfabrication, the prepared primary hepatocyte–endothelial cell heterospheroids maintained albumin secretory capacity for a longer time compared to 2D culture [4]. Furthermore, primary hepatocyte–endothelial cell heterospheroid arrays were significantly superior in albumin secretory capacity, CYP3A activity, and glucuronidation when compared with 2D cultures [4,5,17]. Transmission electron microscopy also revealed a liver-specific Disse lumen structure between endothelial cells and hepatocytes, indicating successful formation of miniature livers [5].

As a safe and minimally invasive diagnostic method, MRI contrast agents are widely used to contrast the condition of the blood vessels, various organs, and lesion areas. In particular, iron- and gadolinium-based contrast agents are expected to produce a high contrast effect through interactions with the surrounding protons. We focused on an MRI contrast agent already approved as a drug, polysaccharide magnetic nanoparticle composite (Resovist^®^; ATDM), and synthesized several types of magnetic nanoparticles with different properties (cationic and anionic groups, particle size, zeta potential, etc.) [27,28,29,30,31]. In addition, we investigated the stability of the synthesized nanoparticles in the culture medium and their cellular uptake. The results showed that cationic polysaccharide magnetic particles (TMADM) effectively take up various cells [27,28,29,30,31]. In particular, TMADM can be labeled on pancreatic islets in a short time and visualized after implantation in mice [29]. Furthermore, transmission electron microscopy of the internal structure of human hepatocellular carcinoma cells (HepG2) monospheroids labeled with TMADM showed that TMADM accumulated in the central and inner cell lysosomes of spheroids [30].

We then examined the morphology of the four synthesized magnetic nanoparticles. For the 3D liver models, we prepared primary hepatocyte–endothelial cell heterospheroids, that is, miniature livers with liver-specific functions. The toxicity, CYP3A, and albumin secretory capacity were evaluated in the heterospheroids labeled with several polysaccharide magnetic iron oxide nanoparticles.

## 2. Materials and Methods

### 2.1. Chemicals

Dulbecco’s modified Eagle’s medium with high glucose, L-glutamine, phenol red, sodium pyruvate (DMEM), William’s E Medium, Dulbecco’s phosphate-buffered saline without Ca^2+^ and Mg^2+^ supplementation (DPBS-free), and antibiotics (penicillin and streptomycin) were obtained from Thermo Fisher Scientific Inc. (Waltham, MA, USA). Fetal bovine serum (FBS; BIO-WEST) was obtained from Funakoshi Co., Ltd. (Tokyo, Japan). Dimethyl sulfoxide (DMSO) (D2650), testosterone (UC339), and 6-β-hydroxytestosterone (UC282) were purchased from Sigma-Aldrich Fine Chemicals (St. Louis, MO, USA). Collagenase (032–10534 for cell isolation) was purchased from Wako Pure Chemicals (Osaka, Japan). All other materials and chemicals used in the study were of the highest grade available.

### 2.2. Polysaccharide Magnetic Iron Oxide Nanoparticles

**Scheme 1:**
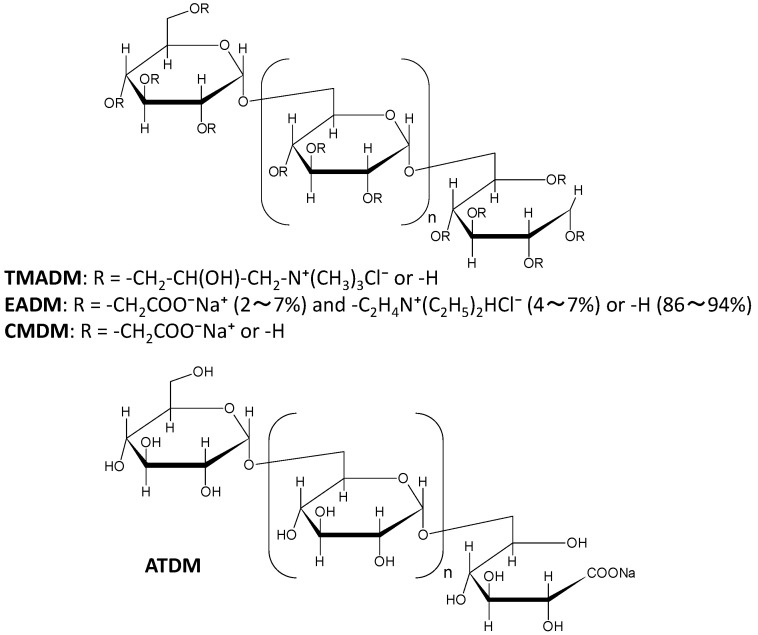
A schematic illustration of the magnetic iron oxide nanoparticles used in this study.

Trimethylamino dextran-coated magnetic iron oxide nanoparticles (TMADM), diethylaminomethyl dextran-coated magnetic iron oxide nanoparticles (EADM), alkali-treated dextran-coated magnetic iron oxide nanoparticles (ATDM), and carboxymethyl dextran-coated magnetic iron oxide nanoparticles (CMDM) were provided by MEITO Sangyo Co., Ltd. (Kiyosu, Japan), as described previously [28,31] (Scheme 1). ATDM was the raw material for the iron-based MRI contrast agent, Resovist^®^.

### 2.3. Size and Zeta Potential Measurement

The average hydrodynamic diameter and the polydispersity index of nanoparticles were determined by dynamic light scattering (DLS) using an ELSZneo (Otsuka Electronics Co., Ltd., Osaka, Japan) fitted with a 638 nm laser beam at a fixed angle of 165°. Measurements were taken at 25 °C, with 0.89 × 10^−2^ Pa.s viscosity and a refractive index of 1.33. The nanoparticles were concentrated at 0.025 mg/mL in 2.5 mM phosphate buffer solution (pH 7.4), to enable measurements (performed in triplicate), in order to assure a convenient scatter intensity on the detectors. Three measurements were carried out for each sample and mean values with standard deviations were calculated. The zeta potential measurements were carried out using a ZetaSizer nano ZS (Malvern Instruments, Malvern, UK). Each sample was properly diluted with 0.025 M phosphate buffer and maintain the temperature at 25 °C.

### 2.4. SEM and TEM Micrographs of Polysaccharide Magnetic Iron Oxide Nanoparticles

The morphology of polysaccharide magnetic iron oxide nanoparticles was observed by a field emission scanning electron microscope (FE-SEM) (Hitachi High-Technologies Co., SU9000) (Tokyo, Japan). Ultrasonically dispersed nanoparticles onto a collodion membrane were observed at an accelerating voltage of 15 kV. The structure and morphology of polysaccharide magnetic iron oxide nanoparticles were observed using a transmission electron microscopy (TEM) (H-7650 type zero A, Hitachi) (Tokyo, Japan). Ultrasonically dispersed nanoparticles onto a collodion membrane were observed at an accelerating voltage of 120 kV.

### 2.5. Cells and Animals

Bovine carotid artery normal endothelial cells (HH) (JCRB0099) were obtained from the Japanese Collection of Research Bioresources Cell Bank (Osaka, Japan). Male Sprague-Dawley rats (6–7 weeks old, specific pathogen-free) weighing approximately 200–250 g were purchased from SLC Japan. The rats were housed under specific pathogen-free conditions with a 12-h light/dark cycle and had free access to food and water. Rat studies were approved by the review committee of Nagoya University Graduate School of Medicine. Rat hepatic parenchymal cells (primary hepatocytes) were prepared from male Sprague–Dawley rats by the collagenase perfusion method [32] Cell viability was >85%, as determined by the trypan blue dye exclusion test using trypan blue stain (Gibco) at a final concentration of 0.2%.

### 2.6. 3D Spheroids Culture of Hepatocytes and Endothelial Cells

The 12-well cell culture plates for 3D spheroids culture (Cell-able^TM^) were supplied by Sumitomo Bakelite (Tokyo, Japan). HH cells (3 × 10^5^) were inoculated onto the cell array and cultured in 1 mL of DMEM containing 10% FBS and antibiotics (100 U/mL penicillin and 100 U/mL streptomycin). Rat hepatic parenchymal cells (primary hepatocyte) (4 × 10^5^/well) were inoculated into the HH-precultured cell array to form heterospheroids. The heterospheroids were cultured in Williams Medium E containing 10% FBS, 1 μmol/L insulin, 1 μmol/L dexamethasone, and antibiotics. The heterospheroids were incubated for two days at 37 °C in an incubator with a humidified 5% CO_2_ atmosphere. The heterospheroids were then incubated with polysaccharide magnetic iron oxide nanoparticles (0, 0.1, 0.5, 1, 5, 15, 30, and 60 µg Fe/mL) for 24 h at 37 °C. Hepatic function and *in vitro* cytotoxicity started 3 d after inoculation of the heterospheroids.

### 2.7. Functional Examination of Hepatocyte–Endothelial Cell Heterospheroids Labeled with Polysaccharide Magnetic Iron Oxide Nanoparticles

Hepatocyte–endothelial cell heterospheroids labeled with polysaccharide magnetic iron oxide nanoparticles were also assayed calorimetrically for metabolic activity using formazan formation (Cell Counting Kit-8 (WST-8); Dojindo Molecular Technologies Inc., Kumamoto, Japan). Rat primary hepatocytes on the cell array were assayed for CYP3A activity by testosterone 6-β-hydroxylation, as described previously [33]. Cytochrome P450 subfamily CYP3A activity was estimated by the rate of testosterone 6-β-hydroxylation. The heterospheroids were incubated with 150 μmol/L testosterone (T1500; Sigma, MO, USA) for 4 h, and the formation of 6-β-hydroxytestosterone was determined by high performance liquid chromatography with a reversed-phase analytical column (TSKgel Super-ODS18197, TOSOH, Tokyo, Japan). Albumin production was assessed by measuring its accumulation in the culture medium for 24 h using a rat albumin ELISA (Enzyme-Linked Immunosorbent Assay) quantification kit according to the manufacturer’s instructions (E111-125; Bethyl Laboratories, Inc., Montgomery, TX, USA).

### 2.8. Statistical Data Analysis

Data are presented as mean ± standard error. Each experiment was repeated at least in triplicates (*n* ≥ 3). Statistical significance was determined using Welch’s *t*-test for comparisons between two groups; Statistical significance was set at *p*-values (*p* < 0.05, and *p* < 0.01).

## 3. Results

### 3.1. Morphology, Size, and Zeta Potential of Polysaccharide Magnetic Iron Oxide Nanoparticles

**Figure 1:**
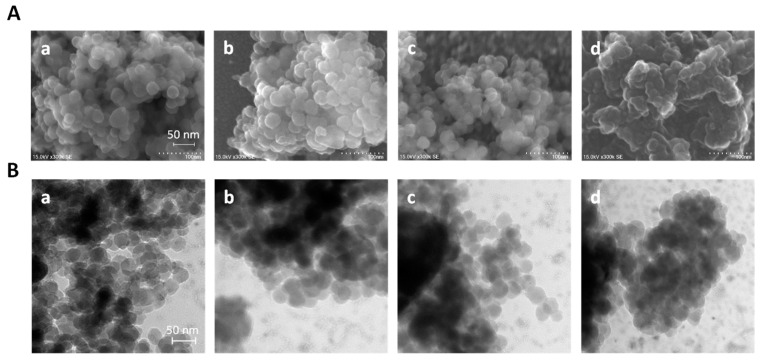
(**A**) SEM micrograph of polysaccharide magnetic iron oxide nanoparticles ((**a**). TMADM, (**b**). EADM, (**c**). ATDM, (**d**). CMDM). Scale bar: 50 nm; (**B**) TEM micrograph of polysaccharide magnetic iron oxide nanoparticles ((**a**). TMADM, (**b**). EADM, (**c**). ATDM, (**d**). CMDM). Scale bar: 50 nm.

We synthesized and evaluated various magnetic polysaccharide iron oxide nanoparticles (Scheme 1), as described previously [28,31]. The morphology of various nanoparticles was observed by FE-SEM and TEM (Figure 1). The morphology of various nanoparticles showed relatively smooth-edged spherical shapes and the aggregate formation by FE-SEM micrograph (Figure 1A). The morphology of various nanoparticles showed nearly spherical shapes and the aggregate formation by TEM micrograph (Figure 1B). The nanoparticle size and zeta potential were measured from dynamic light scattering and Zetasizer, respectively (Table 1). TMADM and EADM were efficiently transduced into cells because of their positive charges [28]. In contrast, the negatively charged nanoparticles ATDM and CMDM were transduced to a lesser extent into the cells [28].

### 3.2. Morphology of Hepatocyte–Endothelial Cell Heterospheroids Labeled with Polysaccharide Magnetic Iron Oxide Nanoparticles

**Figure 2:**
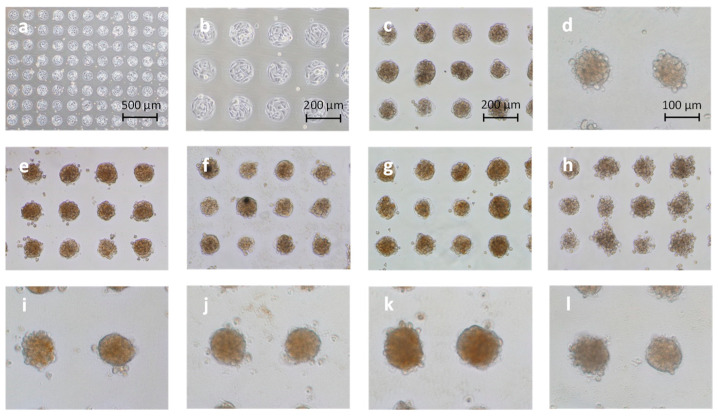
Morphology of 3D liver spheroids labeled with polysaccharide magnetic iron oxide nanoparticles. Endothelial cells (3 × 10^5^) were inoculated for 24 h onto a Cell-able 12-well plate (**a**,**b**). Hepatocytes were seeded (4 × 10^5^/well) and cultured for 48 h on the endothelial monospheroids. After 48 h of cell culture, hepatocyte–endothelial cell heterospheroids were incubated with 60 µg-Fe/mL various nanoparticles in a culture medium for 24 h at 37 °C. The type of nanoparticle: Control (non-labeled) (**c**,**d**); TMADM (**e**,**i**); EADM (**f**,**j**); ATDM (**g**,**k**); CMDM (**h**,**l**). Scale bar: 500, 200, and 100 µm.

Figure 2 shows a phase-contrast photomicrograph of hepatocyte–endothelial cell heterospheroids labeled with various nanoparticles. After creating the endothelial cell monospheroids (Figure 2a,b), the heterospheroids were prepared by seeding primary hepatocytes on the monospheroids. We have previously reported that the prepared hepatocyte–endothelial cell heterospheroids have hepatocyte-specific structures and functions [5,17]. The heterospheroids were incubated with 60 µg-Fe/mL various nanoparticles (TMADM, EADM, ATDM, and CMDM) in a culture medium for 24 h at 37 °C. The morphology color of the heterospheroids labeled with various nanoparticles (Figure 2e–l) is darker compared to the control group (non-labeled heterospheroids) (Figure 2c,d). These results are consistent with those reported for HepG2 cell monospheroids [30,31].

### 3.3. Metabolic Activity of Hepatocyte–Endothelial Cell Heterospheroids

**Figure 3:**
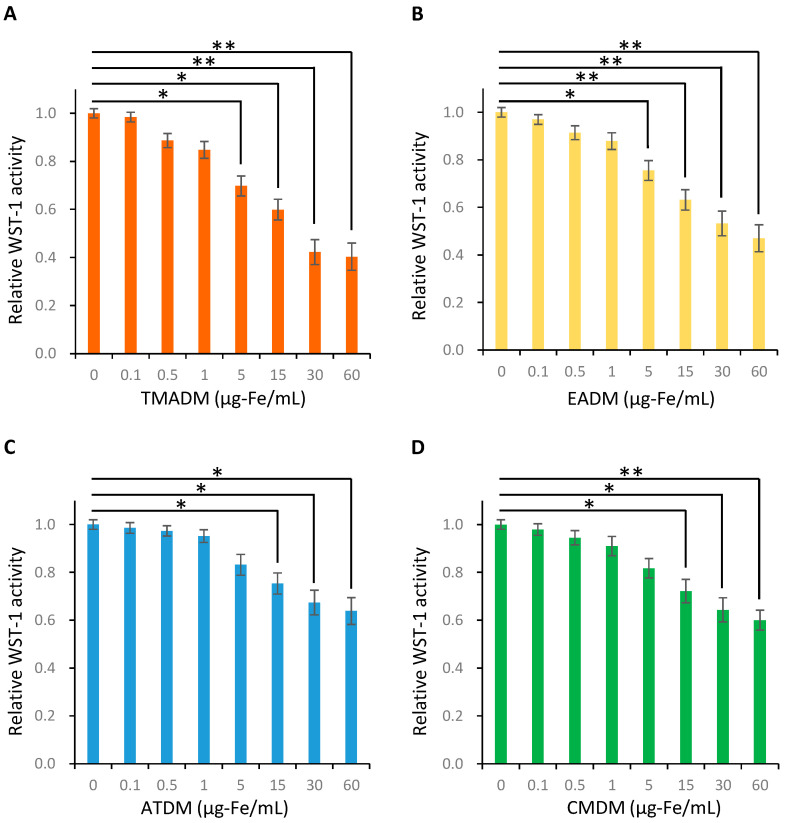
Metabolic activity of hepatocyte–endothelial cell heterospheroids labeled with polysaccharide magnetic iron oxide nanoparticles was estimated by the accumulation of formazan (WST-1 assay). The type of nanoparticle: (**A**) TMADM; (**B**) EADM; (**C**) ATDM; (**D**) CMDM. The heterospheroids were incubated for 2 d at 37 °C. After 48 h of cell culture, the heterospheroids were then incubated with various nanoparticles (0, 0.1, 0.5, 1, 5, 15, 30, and 60 µg-Fe/mL) for 24 h at 37 °C. The assay started 3 d after the inoculation of the heterospheroids (mean ± range, *n* = 5). Relative WST-1 activity of the heterospheroids at each concentration of nanoparticles was converted using the control group (non-labeled heterospheroids, 0 µg-Fe/mL) as the 1.0 value. *p*-values are presented. Welch’s *t*-tests were used to compare WST-1 activity of the control group *vs.* the labeled groups. No mark = no significance, * *p* < 0.05, ** *p* < 0.01.

The metabolic activities of hepatocyte–endothelial cell heterospheroids are shown in Figure 3. The heterospheroids assessed *in vitro* cytotoxicity (the WST-1 assay) of various nanoparticles (Figure 3). As the concentration of nanoparticles increased, the heterospheroids showed a decrease in formazan, a metabolite formed by mitochondrial dehydrogenase. The heterospheroids labeled with a high amount of nanoparticles inhibited cell proliferation. The heterospheroids labeled with anionic nanoparticles (ATDM and CMDM) showed higher activity than the cultured heterospheroids labeled with cationic nanoparticles (TMADM and EADM). Relative WST-1 activity was significantly different in the control group (non-labeled heterospheroids) and the labeled groups (control group *vs.* various nanoparticles (concentration higher than 5 µg-Fe/mL), *p* < 0.05; control group *vs.* TMADM and EADM (concentration higher than 30 µg-Fe/mL), *p* < 0.01).

**Figure 4:**
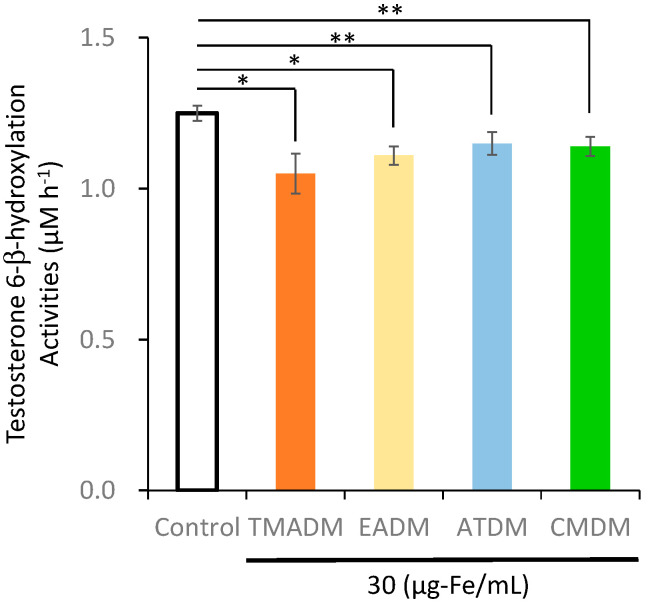
Drug-metabolizing activities of hepatocyte–endothelial cell heterospheroids labeled with polysaccharide magnetic iron oxide nanoparticles (TMADM, EADM, ATDM, and CMDM). The heterospheroids were incubated for 2 d at 37 °C. After 48 h of cell culture, the heterospheroids were then incubated with various nanoparticles (30 µg-Fe/mL) for 24 h at 37 °C. Testosterone 6-β-hydroxylation was determined by 4 h incubation with 100 μmol/L testosterone. The data shown represent the means and standard deviation (SD) of three independent experiments. Statistically significant difference was observed in the control group (non-labeled heterospheroids, 0 µg-Fe/mL) and the labeled groups with various nanoparticles (* *p* < 0.01, ** *p* < 0.05).

Hepatocyte–endothelial cell heterospheroids maintained cytochrome P450-mediated monooxygenation [5]. Cytochrome P450 activity (CYP3A, estimated as testosterone 6-β-hydroxylation) of heterospheroids was decreased with various nanoparticles (Figure 4). The CYP3A activity was significantly different in the control group (non-labeled heterospheroids) and the labeled groups (control group *vs.* TMADM and EADM, *p* < 0.01; control group *vs.* ATDM and CMDM, *p* < 0.05). The heterospheroids labeled with anionic nanoparticles (ATDM and CMDM) showed higher activity than the cultured heterospheroids labeled with cationic nanoparticles (TMADM and EADM).

### 3.4. Albumin Synthesis of Hepatocyte–Endothelial Cell Heterospheroids

**Figure 5:**
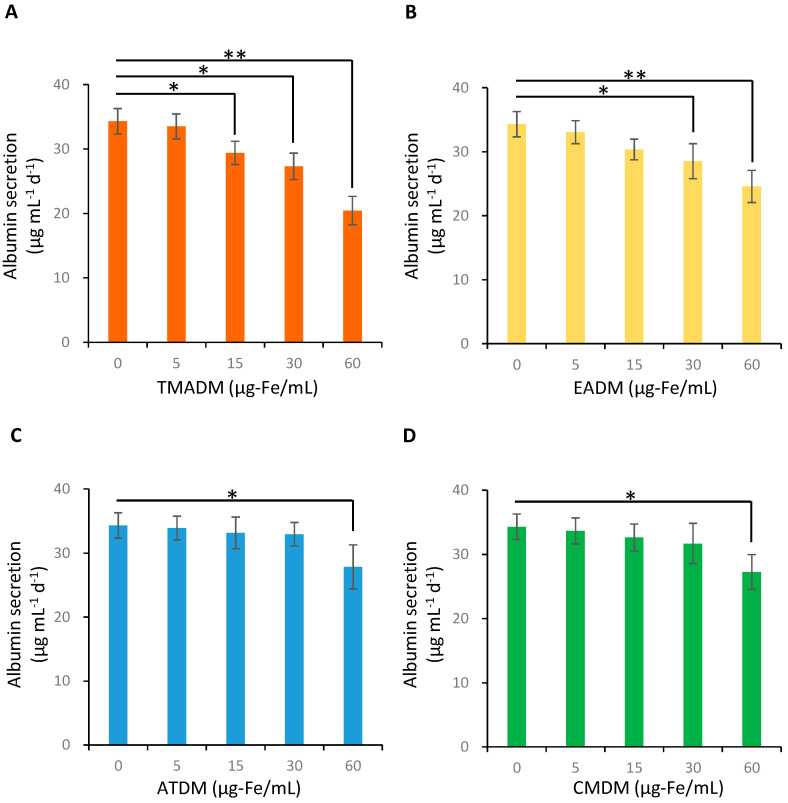
Albumin secretion of hepatocyte–endothelial cell heterospheroids labeled with polysaccharide magnetic iron oxide nanoparticles. The type of nanoparticle: (**A**) TMADM; (**B**) EADM; (**C**) ATDM; (**D**) CMDM. The heterospheroids were incubated for 2 d at 37 °C. After 48 h of cell culture, the heterospheroids were then incubated with various nanoparticles (0, 5, 15, 30, and 60 µg-Fe/mL) for 24 h at 37 °C. The albumin secretion by heterospheroids on day 3 was measured with ELISA. The data shown represent the means and SD of three independent experiments. *p*-values are presented. Welch’s *t*-tests were used to compare albumin secretion of the control group (non-labeled heterospheroids, 0 µg-Fe/mL) *vs.* the labeled groups. No mark = no significance, * *p* < 0.05, ** *p* < 0.01.

**Figure 6:**
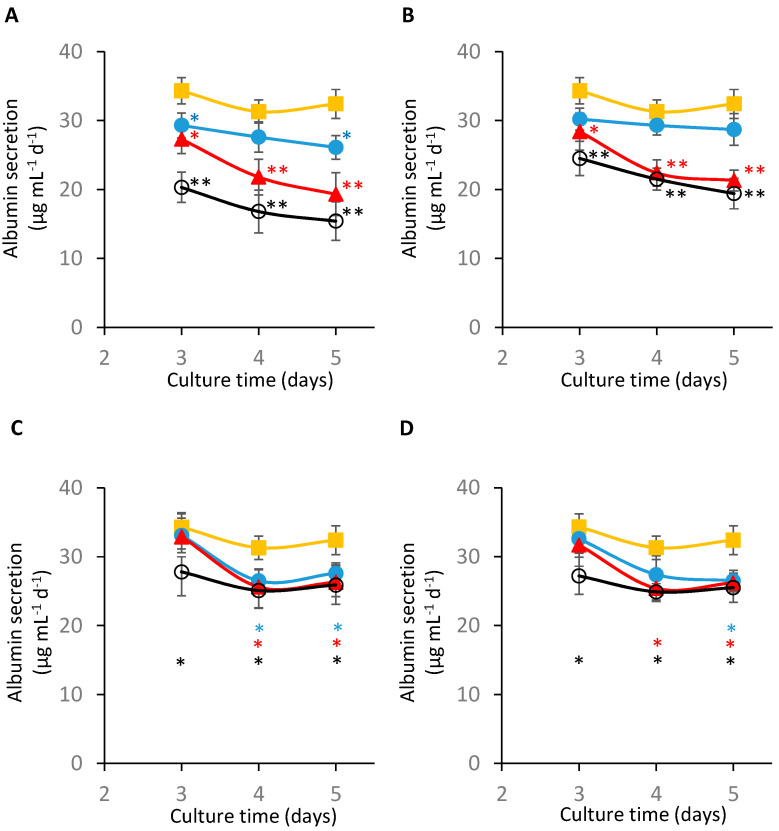
Time course of Albumin secretion of hepatocyte–endothelial cell heterospheroids labeled with polysaccharide magnetic iron oxide nanoparticles. The type of nanoparticle: (**A**) TMADM; (**B**) EADM; (**C**) ATDM; (**D**) CMDM. The heterospheroids were incubated for 2 d at 37 °C. After 48 h of cell culture, the heterospheroids were then incubated with various nanoparticles (0, 15, 30, and 60 µg-Fe/mL) for 24 h at 37 °C. The addition conditions of various nanoparticle: control group (non-labeled heterospheroids, 0 µg-Fe/mL) (closed orange squares, ■); 15 µg-Fe/mL (closed sky-blue circles, ●); 30 µg-Fe/mL (closed red triangles, ▲); 60 µg-Fe/mL (open circles, ◯). The albumin secretion by heterospheroids on days 3, 4, and 5 was measured with ELISA. The data shown represent the means and SD of three independent experiments. *p*-values are presented. Welch’s *t*-tests were used to compare albumin secretion of the control group (non-labeled heterospheroids, 0 µg-Fe/mL) *vs.* the labeled groups. No mark = no significance, * *p* < 0.05, ** *p* < 0.01.

The amount of albumin secreted by hepatocyte–endothelial cell heterospheroids is shown in Figure 5 and Figure 6, respectively. Figure 5 shows the albumin secretion by the heterospheroids on day 3. As the concentration of various nanoparticles increased, albumin secretion of the heterospheroids decreased for various nanoparticles. Albumin secretion was significantly different in the control group (non-labeled heterospheroids) and the labeled groups (control group *vs.* TMADM and EADM (concentration higher than 15 or 30 µg-Fe/mL), *p* < 0.05; control group *vs.* ATDM and CMDM (60 µg-Fe/mL), *p* < 0.05; control group *vs.* TMADM and EADM (60 µg-Fe/mL), *p* < 0.01). The heterospheroids labeled with anionic nanoparticles (ATDM and CMDM) showed higher albumin secretory capacity than the cultured heterospheroids labeled with cationic nanoparticles (TMADM and EADM).

Figure 6 shows the albumin secretion by the heterospheroids on days 3, 4, and 5. Statistically, significant difference was observed in the control group and the labeled groups with 60 µg-Fe/mL various nanoparticles (TMADM and EADM: days 3, 4, and 5 *p* < 0.01; ATDM and CMDM: days 3, 4, and 5 *p* < 0.05). As the culture period progressed, the albumin secretion of the labeled groups decreased significantly (control group *vs.* TMADM and EADM: day 3 (15 or 30 µg-Fe/mL, *p* < 0.05) *vs.* days 4 and 5 (30 µg-Fe/mL, *p* < 0.01); control group *vs.* ATDM and CMDM: day 3 (60 µg-Fe/mL, *p* < 0.05) *vs.* days 4 and 5 (15 or 30 µg-Fe/mL, *p* < 0.05). As the culture period progressed, the heterospheroids labeled with anionic nanoparticles (ATDM and CMDM) showed higher albumin secretory capacity than the cultured heterospheroids labeled with cationic nanoparticles (TMADM and EADM).

## 4. Discussion

It is critical to evaluate the effects of contrast agents and drugs, such as nanoparticles, on cells for purposes of medical and clinical research. We expect to modify the clinical MRI contrast agents (Resovist^®^) and use them as a visualization tool for cell transplantation. The most common compounds utilized in MRI for contrast enhancement are iron oxide and gadolinium [34]. Feridex^®^ is a superparamagnetic iron oxide (SPIO) colloid with low molecular weight dextran with a particle size of 120–180 nm. Resovist^®^ is carboxydextrane-coated SPIO with a particle size of 45–60 nm. Primovist^®^ is gadolinium ethoxybenzyl dimeglumine (Gd-EOB-DTPA) with a molecular weight of 725.71. This study focused on ATDM (Resovist^®^) and evaluated the other iron-based polysaccharide nanoparticles (TMADM, EADM, and CMDM) for labeling target cells. We synthesized and evaluated characteristic features of various nanoparticles (ATDM, TMADM, EADM, and CMDM), as described previously [28,31]. The morphology of magnetic polysaccharide nanoparticles by FE-SEM and TEM showed relatively smooth-edged spherical shapes and nearly spherical shapes, respectively. Similar to previous reports, dextran-based nanoparticles exhibit aggregate formation [35,36]. The particle size of ATDM by TEM or DLS was approximately 23–30 nm and 57.9 ± 0.5 nm, respectively. TEM sizes are smaller than those obtained by DLS. Therefore, TEM determines the actual size of dried nanoparticle formulations. On the other hand, DLS measures the size of ATDM in an aqueous solution. These results are consistent with previous reports by Resovist^®^, who measured the hydrodynamic diameter of Resovist^®^ in an aqueous solution and incorporated the surface-bound water layer in DLS [35,36].

Many researchers have reported numerous comparisons between the 2D and 3D cultures of drugs [1,2,3,5,13,14,15,37,38]. Most importantly, the selection of 3D cultures allows for longer retention of cellular function and better test sensitivity than 2D cultures. Therefore, 3D culture techniques have been widely used in drug discovery, medicine, and cosmetics. The human body is composed of various cells, among which the cells of the liver play an essential role in metabolism, detoxification, bile production, and secretion [4,5,6,7,8,11,12,13,14,15,39,40,41]. In order to investigate the effect of nanomaterials on the liver, a 3D liver model with liver-specific functions must be created *in vitro*. Several studies have been reported on the effects of nanomaterials on 3D liver models [30,42,43,44,45]. Kermanizadeh A. et al. evaluated the hepatotoxicity of human liver cell heterospheroids by various nanomaterials (Ag, ZnO, MWCNT, and a positively charged TiO_2_) [42]. Mekky G. et al. reported on the effects of magnesium oxide nanoparticles using primary human liver cell heterospheroids and animals [43]. Tee J.K. et al. reported on the effects of TiO_2_ nanoparticles using LO2 cell–sinusoidal endothelial cell heterospheroids [44]. Miyamoto Y. et al. reported on the distribution of magnetic polysaccharide nanoparticles in HepG2 cell monospheroids [30]. Fleddermann J. et al. reported on the distribution of SiO_2_ nanoparticles in HepG2 cell monospheroids [45]. Various types of nanoparticles can be evaluated for liver functions in 3D liver models [30,42,43,44,45], and the obtained results can support *in vivo* [43]. In our previous reports, the heterospheroids of endothelial cells and primary hepatocytes exhibited liver-like structures, and maintained the albumin secretory capacity, CYP3A activity, and glucuronidation for an extended period [4,5]. In this study, the heterospheroids were used to investigate the effects of various nanoparticles.

The morphology of the heterospheroids labeled with various nanoparticles (Figure 2) did not differ significantly from that of the HepG2 cell monospheroids labeled with nanoparticles [30]. The heterospheroids labeled with ATDM and CMDM exhibited higher WST-1 activity than that labeled with TMADM and EADM (Figure 3). This result shows that the nanoparticles have anionic and cationic characteristics, respectively (Table 1).

In our previous reports, Yukawa H. et al. reported the viability of adipose tissue-derived stem cells (ASCs) labeled with ATDM or TMADM [46]. The viability of ASCs was significantly different in non-labeled ASCs and ASCs labeled with TMADM (concentration 5–100 µg-Fe/mL, *p* < 0.05). On the other hand, the viability of ASCs was not significantly different in non-labeled ASCs, and ASCs labeled with TMADM (concentration 5–100 µg-Fe/mL). The proliferation rate of ASCs labeled with TMADM (concentration 10–50 µg-Fe/mL) was also maintained compared to non-labeled ASCs. Anionic iron-based polysaccharide nanoparticles (ATDM and CMDM) have been reported to exist primarily near the cell surface in 2D culture [27,30,31,46]. The cationic nanoparticles (TMADM and EADM) are reported to be mainly taken up by cells and accumulate in lysosomes [30,31,46]. The quantitative determinations of the ATDM and TMADM internalized into ASCs by measuring the amount of Fe derived from ATDM or TMADM using ICP-AES [46]. The amount of Fe in cells labeled with TMADM was about four-fold higher than that of ATDM. No significant differences in cell function were observed compared to ASCs labeled with ATDM and non-labeled ASCs.

However, WST-1 activity significantly differed between heterospheroids labeled with anionic nanoparticles (ATDM and CMDM) and non-labeled heterospheroids (Figure 3C,D). The CYP3A activity significantly differed between heterospheroids labeled with various nanoparticles and non-labeled heterospheroids (Figure 4). We evaluated the albumin secretory capacity of the heterospheroids labeled with various nanoparticles compared to non-labeled heterospheroids. Albumin secretion was significantly different between the heterospheroids labeled with various nanoparticles and non-labeled heterospheroids on day 3 (60 µg-Fe/mL, *p* < 0.05) (Figure 5). As the culture period progressed, the albumin secretion of the heterospheroids labeled with various nanoparticles decreased significantly compared to non-labeled heterospheroids (day 5, 15 µg-Fe/mL, *p* < 0.05). The albumin secretion of heterospheroids labeled with anionic nanoparticles (ATDM and CMDM) compared to non-labeled heterospheroids were significantly different between day 3 and day 5 (day 3, 60 µg-Fe/mL, *p* < 0.05; day 5, 15–60 µg-Fe/mL, *p* < 0.05).

In previous reports, Kermanizadeh A. et al. evaluated the toxicity and hepatic function of human liver cell heterospheroids (hepatocyte and non-parenchymal cells) by exposure to various nanomaterials (Ag, ZnO, MWCNT, and a positively charged TiO_2_) [42]. The repeated exposure to nanomaterials for the heterospheroids was more toxic than a single exposure. The cytotoxicity and viability of the heterospheroids were markedly reduced by exposure to nanomaterials. On the other hand, there was no significant difference in albumin secretion of heterospheroids by nanomaterial exposure. Albumin is a protein produced by the liver and is a standard indicator of liver function. These differences in albumin secretion of the heterospheroids may depend on the type of nanomaterials and various cells. Tee J.K. et al. created the 3D liver models (heterospheroids) using LO2 cells and sinusoidal endothelial cells [44]. The structure of these heterospheroids (Tee J.K. et al. [44]) is similar to that of our created heterospheroids (Enosawa S. and Miyamoto Y. et al. [5]). Because the hepatocyte is the core of the heterospheroids, and the endothelial cells are located on the outside heterospheroids. As the concentration of TiO_2_ nanoparticles increased, the sinusoidal endothelial cells in the heterospheroids decreased compared to the controls (heterospheroids without TiO_2_ nanoparticles) [44]. TiO_2_ nanoparticles weakened the adhesion between the sinusoidal endothelial cell layers and the core of the hepatocytes in the heterospheroids and detached the sinusoidal endothelial cells in the heterospheroids [44]. In our previous reports, Enosawa S. and Miyamoto Y. et al. reported that the albumin secretion in the hepatocyte–endothelial cell heterospheroids was approximately 2-fold higher than in hepatocyte monospheroids in short-term cultures [5]. In this study, we also showed that the albumin secretion of hepatocyte–endothelial cell heterospheroids labeled with TMDM (day 5, 60 µg-Fe/mL) was approximately 2-fold lower than that of non-labeled heterospheroids (day 5, 0 µg-Fe/mL) (Figure 6A). The decrease in albumin secretion may be due to detaching the outer endothelial cells of the heterospheroids by labeling the nanoparticles.

In this study, we focused on anionic polysaccharide magnetic iron oxide nanoparticles (Resovist^®^) and synthesized four types of magnetic iron oxide nanoparticle derivatives with different properties. In our previous reports, the ASCs labeled with TMADM (≥30 mg-Fe/mL) could be detected by MRI imaging and could be traced for at least 14 days after transplantation with the skin in the mice [46]. Therefore, we expect to modify the clinical MRI contrast agents and use them as a visualization tool for artificial 3D liver transplantation. However, the liver function of hepatocyte–endothelial cell heterospheroids labeled with various nanoparticles decreased even at low concentrations (≥5 or 15 mg-Fe/mL). Possible causes are (1) primary hepatocyte damage and (2) detachment of endothelial cells outside the heterospheroids [44] with the use of nanoparticles. To create an artificial 3D liver, the selection of cell sources is essential, including primary cells [47,48], immortalized cells [49], lineage cells, and stem cells [50,51]. Human hepatic parenchymal and nonparenchymal cells are difficult to obtain due to a lack of donors. The hepatocytes differentiated from induced pluripotent stem cells (iPSCs) [52,53,54,55], embryonic stem cells (ESCs) [56], and tissue stem cells [57] are expected to be a new cell source. Recently, *in vitro* 3D culture techniques have been used to create liver and multi-organ models [6,58]. Koike H. and Takebe T. et al. reported a technique for continuously creating liver, bile, and pancreas (HBP) structures from 3D cultures of human PSCs [58]. Peng W.C. and Nusse R. et al. have successfully cultured hepatocytes for more than six months in 3D culture [59]. These hepatocytes grown in 3D culture were transplanted into mice livers and significantly repopulated [59]. In the future, there is a need to improve the method of creation of artificial 3D liver or to design a low-invasive MRI contrast agent to label the artificial 3D liver.

## 5. Conclusions

In this study, we examined the morphology of the four synthesized polysaccharide magnetic iron oxide. The morphology of various nanoparticles showed relatively smooth-edged spherical shapes. For the 3D liver models, we prepared primary hepatocyte–endothelial cell heterospheroids. The toxicity, CYP3A, and albumin secretory capacity were evaluated in the heterospheroids labeled with various nanoparticles. As the culture period progressed, the heterospheroids labeled with anionic and cationic nanoparticles showed lower liver function than non-labeled heterospheroids. In the future, there is a need to improve the method of creation of artificial 3D liver or to design a low-invasive MRI contrast agent to label the artificial 3D liver.

## Figures and Tables

**Table 1:** Characteristic features of synthesized polysaccharide magnetic iron oxide nanoparticles.

Name	Diameter (nm)	Polydispersity Index	Zeta Voltage (mV)
TMADM	51.5 ± 0.6	0.157 ± 0.005	+32.8
EADM	52.1 ± 0.9	0.256 ± 0.007	+30.1
ATDM	57.9 ± 0.5	0.349 ± 0.046	−14.7
CMDM	46.0 ± 1.2	0.231 ± 0.011	−23.6

## Data Availability

Not applicable.
